# An update on semen quality among young Finnish men and comparison with Danish data

**DOI:** 10.1111/andr.12550

**Published:** 2018-09-24

**Authors:** W. Rodprasert, H. E. Virtanen, S. Sadov, A. Perheentupa, N. E. Skakkebæk, N. Jørgensen, J. Toppari

**Affiliations:** ^1^ Research Centre for Integrative Physiology and Pharmacology Institute of Biomedicine University of Turku Turku Finland; ^2^ Department of Obstetrics and Gynecology University of Turku and Turku University Hospital Turku Finland; ^3^ University Department of Growth and Reproduction and EDMaRC Rigshospitalet Copenhagen Denmark; ^4^ Department of Pediatrics Turku University Hospital Turku Finland

**Keywords:** fertility, reproductive health, semen quality, sperm, testis

## Abstract

**Background:**

Finnish men used to have higher semen quality than Danish men. However, recent studies showed that semen quality in Finland has declined, but it has been relatively stable in Denmark.

**Objective:**

This study aimed to compare new data on semen quality of the young Finnish men to that of Danish men.

**Materials and methods:**

In this cross‐sectional study, 18‐ to 19‐year‐old men residing in Turku, Finland and Copenhagen, Denmark, were invited to participate in 2008–2011. Each man filled in a questionnaire, provided one semen sample and underwent andrological examination. Semen samples were analyzed according to WHO. Multiway ANOVA was used to adjust semen variables for duration of sexual abstinence and age (and time from ejaculation to the start of semen analysis for sperm motility).

**Results:**

Altogether 287 Finnish men and 873 Danish men participated in the study. The adjusted median sperm concentrations were 49 and 47 million/mL for Finnish and Danish men, respectively (*p* = 0.48). The adjusted median total sperm counts were 148 million in Finland and 146 million in Denmark (*p* = 0.87). The adjusted median percentages of morphologically normal spermatozoa were 6.9% in Finland and 6.5% in Denmark, *p* = 0.27. Finnish men had higher adjusted median percentages of motile spermatozoa (A+B+C) than Danish men (80% vs. 69%, *p* < 0.001). The proportion of men who had low semen quality (sperm concentration, percentage of morphologically normal spermatozoa or percentage of progressively motile spermatozoa below WHO reference limits) was lower in Finland (25.4%) than in Denmark (34.6%), *p* = 0.004.

**Discussion:**

Considerable percentage of men in both countries had low semen quality. The deteriorating semen quality in Finland may result in decreasing fecundity, which is a cause of concern.

**Conclusion:**

The formerly high semen quality in Finland has converged to the lower Danish levels. Our findings demonstrate the importance of continuing surveillance of semen quality.

## Introduction

Studies have shown geographical variation of semen quality (Carlsen *et al*., 1992; Jørgensen *et al*., 2001, 2002; Punab *et al*., 2002; Swan *et al*., 2003; Levine *et al*., 2017; Virtanen *et al*., 2017). In the Nordic and Baltic regions, the East‐West gradient of semen quality was demonstrated (Jørgensen *et al*., 2002). Sperm concentrations of the young men from general population in Finland, Estonia, and Lithuania were higher than that of the men in Norway and Denmark (Jørgensen *et al*., 2002; Punab *et al*., 2002). Semen quality of the young Swedish men was in the middle of this gradient (Richthoff *et al*., 2002). In addition to the difference in the semen quality, men in Finland and Denmark also have shown differences in other aspects of male reproductive health. Finland has had a low incidence of testicular cancer compared with Denmark, which is among the countries with the highest incidences (Adami *et al*., 1994; Chia *et al*., 2010; Engholm *et al*., 2010; Znaor *et al*., 2014). Congenital cryptorchidism (Boisen *et al*., 2004) and hypospadias (Virtanen *et al*., 2001; Boisen *et al*., 2005) were also reported to be more common in Denmark than in Finland.

At an international level, significant recent trends have been seen with regard to human semen quality and testicular cancer. Semen quality has been shown to have declined in many areas (Levine *et al*., 2017), and the incidence of testicular cancer has been increasing (Znaor *et al*., 2014). These findings have also been observed among Finnish men (Jørgensen *et al*., 2011). In contrast, a recent study of young men in Denmark revealed that semen quality in Denmark has been relatively stable between 1996 and 2016 (Priskorn *et al*., 2018). To investigate these trends further, we have conducted a semen quality study in the young men from general population in these two countries by using the same standardized research protocol.

## Material and Methods

### Study population

This is a cross‐sectional study in the young men in two Nordic countries—Denmark and Finland. The study was conducted at two sites—Institute of Biomedicine, University of Turku in Turku, Finland, and Department of Growth and Reproduction, Rigshospitalet in Copenhagen, Denmark. The man and his mother were born in the country of investigation, and the men were raised there.

In Finland, men who were 18–19 years of age during the study period and residing in Turku, Kaarina or Raisio area in the Southwest Finland were invited to participate in this study, irrespective of their health status. List of the men and their addresses were obtained from the registration authority according to their birth years. The invitation letters were sent to their home addresses. The men were asked to send the reply letters stating their wish to participate or not to participate in the study. In a case of refusal to participate, they were asked to fill in the form about basic characteristics, including height, weight, current home address, and occupation. In Denmark, 18‐ to 19‐year‐old men who had no severe or chronic diseases undergo obligatory medical examination for the military recruitment. Some men postponed the examination until their studies were completed. In collaboration with the military health authority, all of the men who lived and attended the medical examinations in the greater Copenhagen area during the study period were invited to participate in this study. They were invited on the day of examinations regardless of whether they were fit for military service. The recruitment period was from 2009 to 2011 in Turku and autumn 2008–2011 in Copenhagen. In Turku, 289 Finnish men participated in the study and 287 (99.3%) of them provided semen samples. In Copenhagen, of 882 Danish participants, 873 (99.0%) provided semen samples. Participation rates were 9% in Finland and 35% in Denmark.

The study protocol was the same as in the previous semen quality studies of the young men in Europe with the same research protocol for both Denmark and Finland (Jørgensen *et al*., 2002; Punab *et al*., 2002; Paasch *et al*., 2008).

### Questionnaire

Standardized questionnaire was created in English and translated into Danish and Finnish (Jørgensen *et al*., 2002). It was subsequently back‐translated into English to check for consistency. The questionnaire was sent to home address of the participant before the study visit. The questionnaire included questions about general health, maternal history during pregnancy, childhood and pubertal history, education, medical conditions, diseases of reproductive system and treatments, fertility, diet, lifestyle and medications and substance use. The men were requested to ask their mothers to help answer the questions about their pregnancy and childhood history of the men.

On the visit day, the man came to the research center, returned the completed questionnaire, underwent physical examination and provided a semen sample.

### Physical examination

The examination was performed by trained physicians, ten examiners in Denmark and three in Finland. Height and weight were measured. Body hair and the presence of gynecomastia were evaluated. Pubic hair was assessed according to Tanner staging system (Marshall & Tanner, 1970). Testicular size was measured by using Prader orchidometer. Male genitalia were examined and the abnormalities were noted.

### Semen analysis

Participants were asked to abstain from ejaculation for at least 48 h before collecting semen samples. However, semen samples collected following less than 48 h of sexual abstinence were also accepted and included in the analysis. The abstinence period was calculated from the time difference between previous and current ejaculation as reported by the men. Each man provided one sample by masturbation in the private room near the andrology laboratory. In Finland, semen sample collection at home was an alternative for the men who were unwilling to collect samples at the laboratory. In this case, a research nurse sent a container to the participant's home address prior to the visit day. The sample had to be handled carefully to avoid extreme temperature or any spillage and had to be delivered to the laboratory within one hour.

Semen samples were analyzed for semen volume, sperm concentration, sperm motility, and sperm morphology. There were three technicians who analyzed semen samples in Denmark. In Finland, one technician analyzed semen volume, sperm concentration, and sperm motility, and the other person analyzed sperm morphology.

When the semen sample was delivered to the andrology laboratory, the sample was kept in 37°C incubator during liquefaction. Subsequently, semen volume was estimated from the weight of the semen by assuming that sperm density is 1 g/mL (Auger *et al*., 1995). Semen weight was calculated by subtracting weight of empty container from the weight of the same container including semen sample. Sperm motility was assessed at 37°C with heated microscope stage at ×200 magnification. The sperm motility was categorized as grade A, B, C, or D (World Health Organization, 1992, 1999). The percentage of motile spermatozoa (grade A+B+C) and progressively motile spermatozoa (grade A+B) were reported. For the assessment of sperm concentration, samples were diluted in a solution of 0.6 mol/l NaHCO3 and 0.4% (v/v) formaldehyde in distilled water before assessment. Bürker‐Türk haemocytometer (Paul Marienfeld GmbH & Co. KG, Lauda‐Königshofen, Germany) was used in Copenhagen, and improved Neubauer haemocytometer was used in Turku. Semen was smeared, air‐dried, and fixed in 95% ethanol before Papanicolaou staining. Sperm morphology was assessed according to the strict criteria (Menkveld *et al*., 1990; World Health Organization, 2010).

External quality control for sperm concentration was performed during the study period. Center in Copenhagen sent five blinded semen samples to Turku four times per year. The technicians in both countries independently checked the same samples. The results showed that the sperm concentration assessed by the center in Turku was not significantly different from that assessed by the center in Copenhagen (*p* = 0.77).

The data of the Danish men had been published previously as a part of larger studies (Jørgensen *et al*., 2012; Priskorn *et al*., 2018). We included Danish data in the statistical analysis to directly compare the results between countries, as the study protocols and semen analyses were identical.

The study protocol was approved by the institutional review board and local ethics committee. Study was conducted according to the Helsinki II Declaration. All participants provided written informed consent prior to participation. All of the participants received financial compensation, 50 Euro in Finland and 500 DKK (approximately 67 Euro) in Denmark.

### Statistical analysis

The characteristics of the participants were reported by using the mean (SD) or median (5^th^ and 95th percentile) for continuous variables and percentages for categorical variables. Between‐group differences of the participants’ characteristics were analyzed by Pearson chi‐squared or Fisher's exact test in case of categorical variables and Student's t‐test or Mann–Whitney *U*‐test for continuous variables.

Multiway ANOVA was used to adjust semen parameters to the confounding factors and to compare the results between countries, both the semen results from all men and from a subgroup of men who did not take any medications or androgenic hormones, without any past or current andrological diseases, or fertility problem. Age, season, and abstinence time were tested for confounding effect on all semen variables. In addition, the effect of time from ejaculation to start of semen analysis was also tested for the effect on sperm motility. Semen variables were transformed to correct for non‐normal distribution of residuals. Semen volume was ln‐transformed. Cubic root transformation was used for sperm concentration, total sperm count, total number of morphologically normal spermatozoa, and total number of progressively motile spermatozoa. Square root transformation was used for percentage of spermatozoa with normal morphology. Sperm motility was logit‐transformed. Age had a positive confounding effect on semen volume and total number of morphologically normal spermatozoa. Abstinence period had increasing effect on semen volume, sperm concentration, total sperm count, and sperm motility. Therefore, both age and abstinence period were entered into final models as continuous variables. Abstinence period was entered as three continuous variables (three linear splines): 0–48, 49–96, and more than 96 h. Percentage of morphologically normal spermatozoa was not influenced by any of these confounding variables. Season of examination did not have any effect on semen variables and was therefore not included in the final model. Time from ejaculation to the start of semen analysis was entered into the model as two continuous variables (two linear splines): 0–30 min and more than 30 min. The duration of more than 30 min had a negative confounding effect on sperm motility and was also included in the final models of sperm motility. The interlaboratory difference of sperm concentration assessment was not included in the final model. The estimated median semen values were reported at a mean age, a mean abstinence period and at mean time from ejaculation to the start of semen analysis. Analysis of the external quality control for the assessment of sperm concentration and the subgroup analysis of men with no andrological problem and no medication use was also performed by using multiway ANOVA.

Two‐sided *p*‐values of <0.05 were considered statistically significant. For the main outcomes, which are the 8 comparisons of the semen variables between the two countries, the Bonferroni adjusted alpha levels of 0.006 per test (0.05/8) were used. IBM SPSS Statistics v. 24.0 (IBM, Armonk, NY) was used for statistical analyses.

## Results

Characteristics of the participants are shown in Table 1. Finnish men were slightly younger, had a larger testis size, and used less alcohol than Danish men. Similar proportion of men from both countries reported medical health problems, except for differences in the rate of thyroid disease and sexually transmitted diseases. Thyroid disease was reported by three (1%) men in Finland but none in Denmark. Higher percentage of men in Denmark reported a history of sexually transmitted infection. In Finland, 2.4% of men reported history of Chlamydial infection and none reported gonococcal infection. In Denmark, 10.3% of men had had Chlamydial infection and 0.6% had had gonococcal infection. Three Finnish men (1%) were operated for cryptorchidism. None received hormonal therapy. Fourteen Danish men (1.6%) were treated by operation, and three men (0.3%) received hormonal treatment for cryptorchidism. The clinical examination showed similar findings in both countries, except varicocoele was detected more often in the Finnish men than in the Danish men. Gynecomastia was not detected in any men. Higher proportion of the men in Denmark than in Finland had history of maternal and paternal cigarette smoking during pregnancy.

**Table 1:** Characteristics of the participants and self‐reported medical conditions

Characteristic	Median (5th–95th percentile) or percentages		*p*‐value
	Finland (*n* = 287)	Denmark (*n* = 873)	
Age (years)	18.7 (18.5–19.1)	19.1 (18.4–23.1)	<0.001^a^
Height (m)	1.80 (1.70–1.91)	1.81 (1.70–1.92)	0.003^a^
Weight (kg)	73.3 (58.5–99.4)	73.2 (60.3–96.9)	0.65^a^
Body mass index (kg/m^2^)	22.6 (18.4–30.5)	22.4 (18.8–28.7)	0.07^a^
Tanner stage of pubic hair			
IV	3.5%	0.8%	0.01^b^
V	11.2%	12.1%	
VI	85.3%	87.1%	
Testis size by orchidometer (ml)^e^	24	21	<0.001^a^
Year of examination (%)			
2008	0.0%	15.3%	<0.001^b^
2009	44.4%	35.4%	
2010	29.9%	35.5%	
2011	25.7%	13.7%	
Season of examination (%)			
Spring	31%	34%	<0.001^b^
Summer	17%	8%	
Autumn	22%	46%	
Winter	30%	12%	
Duration of education (years)	11.6	12.6	<0.001^d^
Cigarette smokers (%)^f^	45.0%	49.5%	0.19^b^
Smoking (cigarettes/day), all men (mean (SD))	3.3 (5.7)	4.2 (6.5)	0.12^d^
Smoking (cigarettes/day), only smokers (mean (SD))	7 (1–20)	10 (1–20)	0.52^a^
Duration of smoking (years)^g^	2.0 (0.0–6.0)	2.0 (0.0–7.0)	0.56^a^
Alcohol consumption during past week (units)	6 (0–36)	11 (0–42)	<0.001^a^
Diagnosed as having			
Asthma (%)	9.8%	10.9%	0.66^c^
Diabetes (%)^h^	0.7%	0.0%	0.06^c^
Thyroid disease (%)	1.0%	0.0%	0.02^c^
Varicocele (%)	0.3%	0.7%	1.00^c^
Hydrocele (%)	1.4%	1.5%	1.00^c^
Testicular torsion (%)	1.0%	1.5%	0.77^c^
Hypospadias (%)	0.0%	0.6%	0.34^c^
Epididymitis/orchitis (%)	0.3%	0.9%	0.47^c^
Cystitis (%)	4.4%	4.0%	0.82^c^
Prostatitis (%)	0.8%	0.1%	0.14^c^
Sexually transmitted diseases^i^ (%)	2.1%	10.7%	<0.001^c^
Pregnancy history			
Maternal smoking during pregnancy^j^ (%)	11.9%	24.1%	<0.001^b^
Paternal smoking during pregnancy (%)	31.9%	42.5%	0.01^b^
Preeclampsia (%)	2%	2.3%	0.62^b^
Hypertension during pregnancy (%)	4.4%	4%	0.95^b^
Gestational diabetes mellitus (%)	1.6%	0.4%	0.11^c^
Clinical examination			
Testicular position (%)			0.64^c^
Scrotal	100%	99.2%	
Inguinal (one testis or both)	0%	0.3%	
Non‐palpable (one testis or both)	0%	0.5%	
Epididymis abnormality (%)	2.1%	1.6%	0.60^c^
Varicocele (%)	20.6%	12.8%	0.004^c^
Hydrocele (%)	0.7%	0.7%	1.000^c^
Treated for			
Testicular cancer	0.0%	0.1%	1.00^c^
Varicocele	0.3%	0.3%	1.00^c^
Hydrocele	1.4%	1.3%	1.00^c^
Cryptorchidism^k^	1.0%	1.9%	0.44^c^

a Student's *t*‐test.

b Chi‐squared test.

c Fisher's exact test.

d Mann–Whitney *U*‐test.

e Average size of both testes.

f Including both current smokers and ex‐smokers.

g Duration of smoking among cigarette smokers.

h Type 1 or type 2 diabetes.

i Included gonococcal and chlamydial infection.

j Including maternal cigarette smoking of any duration.

k Treatment for cryptorchidism were either surgery or hormones.

Results of the semen variables are reported in Table 2. Adjusted semen volume, sperm concentration, total sperm count, and percentage and total number of morphologically normal spermatozoa were not significantly different between the two countries. Danes had lower sperm motility than Finns. Azoospermia was identified in 3 (1%) Finnish men and 8 (0.9%) Danish men (*p* = 0.85). There were 8.8% of Finnish men and 11.2% of Danish men who had sexual abstinence period less than 48 h, *p* = 0.26. Subgroup analysis of the men who did not take any medications, without any past or current andrological diseases, or fertility problem (trying to conceive for more than 12 months without success) is also shown in Table 2. The results from this subgroup analysis had the same direction as the analysis of all men.

**Table 2:** Semen quality of the young men in Finland and Denmark

Semen parameters	All men (Finland: *n* = 287, Denmark: *n* = 873)				Subgroup^c^ (Finland *n* = 166, Denmark: *n* = 465)			
	Observed		Adjusted^a^	*p*‐value^b^	Observed		Adjusted^a^	*p*‐value^b^
	Mean (SD)	Median (5^th^‐95^th^)	Median (95%CI)		Mean (SD)	Median (5^th^‐95^th^)	Median (95%CI)	
Semen volume (mL)								
Finland	3.2 (1.6)	3.1 (0.9–6.4)	2.8 (2.7–3.0)	0.04	3.1 (1.5)	3.1 (0.8–6.2)	2.7 (2.5–2.9)	0.05
Denmark	3.4 (1.6)	3.2 (1.3–6.2)	3.1 (3.0–3.2)		3.3 (1.5)	3.1 (1.2–6.2)	3.0 (2.8–3.1)	
Sperm concentration (×10^6^/mL)								
Finland	59 (43)	48 (9–139)	49 (44–54)	0.48	61 (42)	51 (11–139)	53 (46–61)	0.66
Denmark	61 (53)	48 (4–167)	47 (44–50)		64 (53)	52 (5–166)	51 (47–56)	
Total sperm count (×10^6^)								
Finland	177 (147)	146 (14–450)	148 (132–165)	0.87	175 (128)	151 (17–454)	147 (128–169)	0.78
Denmark	197 (172)	146 (13–541)	146 (137–156)		202 (171)	157 (15–562)	151 (139–163)	
Percent normal morphology (%)								
Finland	7.5 (4.3)	6.5 (1.5–16.0)	6.9 (6.3–7.5)	0.27	7.7 (4.3)	7.0 (2.0–16.0)	6.9 (6.2–7.7)	0.23
Denmark	7.5 (4.8)	7.0 (0.5–16.0)	6.5 (6.2–6.8)		7.5 (4.8)	7.0 (0.5–16.0)	6.4 (6.0–6.8)	
Percent A+B+C sperm motility (%)								
Finland	77 (16)	82 (40–92)	80 (78–81)	<0.001	78 (15)	83 (48–92)	80 (78–82)	<0.001
Denmark	67 (14)	70 (40–87)	69 (68–70)		67 (14)	69 (39–87)	69 (67–70)	
Percent A+B sperm motility (%)								
Finland	68 (18)	75 (21–86)	70 (68–72)	<0.001	69 (18)	75 (21–86)	71 (68–73)	<0.001
Denmark	57 (16)	59 (28–79)	57 (56–59)		57 (16)	59 (27–79)	57 (56–59)	
Total number of morphologically normal spermatozoa (×10^6^)								
Finland	14 (17)	10 (0–43)	11 (9–13)	0.38	14 (15)	9 (0–43)	10 (9–13)	0.69
Denmark	17 (21)	10 (0–60)	10 (9–11)		18 (21)	10 (0–64)	10 (9–11)	
Total number of A+B motile spermatozoa (×10^6^)								
Finland	132 (114)	104 (3–336)	106 (94–120)	0.001	128 (99)	103 (4–337)	103 (89–119)	0.04
Denmark	119 (109)	89 (5–342)	84 (78–90)		121 (108)	91 (6–344)	86 (78–94)	
Abstinence duration (h)								
Finland	69 (24)	65 (39–117)		0.02^d^	67 (22)	63 (37–112)		0.001^d^
Denmark	74 (34)	63 (36–137)			76 (34)	65 (36–135)		
Time from ejaculation to analysis (min)								
Finland	45 (21)	45 (20–80)		0.003^d^	45 (22)	45 (20–80)		0.02^d^
Denmark	41 (22)	35 (15–85)			40 (23)	35 (15–90)		

a Semen parameters were adjusted for abstinence duration and age. In addition, percentages of sperm motility were also adjusted for the time from ejaculation to start of semen analysis. The adjusted estimates of the analysis of all men represented men at the mean age of 19.4 years with the mean abstinence duration of 73 h and mean time from ejaculation to start of semen analysis of 42 min.

b *p*‐Value shown here is the *p*‐value of the differences of the adjusted semen parameters between the two countries.

c Subgroup analysis included men who did not take any medications, androgenic hormones, without any past or current andrological diseases, or fertility problem (trying to conceive for more than 12 months without success).

d Student's *t*‐test. *p*‐Values are the differences of mean levels between countries.

**Figure 1:**
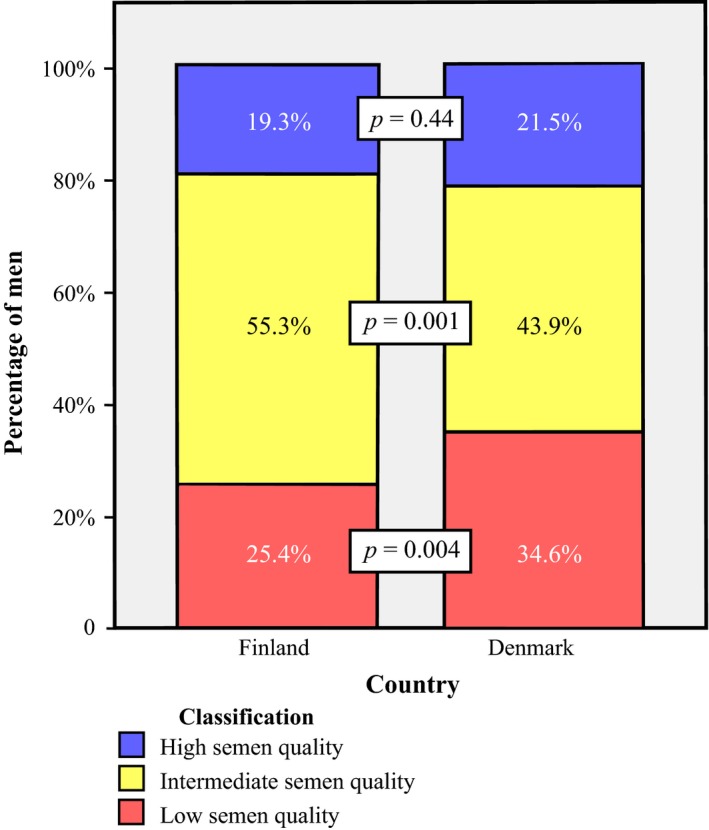
Proportion of men classified as having low, intermediate, or high semen quality based on unadjusted sperm concentration, progressive sperm motility, and percentage of morphologically normal spermatozoa. Low semen quality is defined as sperm concentration <15 million/mL or progressive (A+B) sperm motility <32% or morphologically normal spermatozoa <4% or as any combination of these. High semen quality is defined as sperm concentration >40 million/mL and progressive (A+B) sperm motility >50% and morphologically normal spermatozoa >9%. Other semen quality results are classified as ‘intermediate semen quality’. *p*‐values are the between‐country differences tested by chi‐squared test.

Distribution of semen quality based on the WHO lower reference limits is shown in Table 3 (Cooper *et al*., 2009; World Health Organization, 2010). More men in Denmark had sperm concentration less than 15 × 10^6^/mL than in Finland (15.0% and 9.1%, respectively, *p* = 0.01). Proportion of men who had sperm concentration less than 40 million/mL in Finland was 38.6% and that in Denmark was 41.5%, *p* = 0.39. More men in Finland used medications during 3 months prior to the examination than men in Denmark. When the analysis of drug use classified according to the drug groups was made, similar proportion of men in both countries used each drug class‐oral antibiotics, analgesics, antipyretics, antihistamines, inhalers for asthma or allergic rhinitis, psychiatric and CNS‐acting medications and other medications (data not shown). However, the use of topical medications was more common in Denmark (16%) than in Finland (9%). There were one Finnish man and four Danish men who used supplemental androgenic hormones during three months before the study. Fig. 1 illustrates semen quality of the participating men classified into three categories—low, intermediate, and high semen quality—when sperm concentration, percentage of morphologically normal spermatozoa, and percentage of progressively motile spermatozoa were taken into account together as described previously (Damsgaard *et al*., 2016).

**Table 3:** Proportions of men who had spermatozoa parameters less than WHO lower reference value

Semen parameters	Finland (%)	Denmark (%)	*p*‐value^a^
Sperm concentrations <15 × 10^6^/mL	9.1	15.0	0.01
Total sperm number <39 × 10^6^/ejaculate	12.6	14	0.56
Percentage of morphologically normal spermatozoa < 4%	19.6	25.5	0.05
Percentage of total (A + B + C) sperm motility < 40%	4.6	4.9	0.84
Percent of progressive AB sperm motility < 32%	7.1	6.7	0.80

a Chi‐squared test.

In Finland, a total of 104 men (3.4% of the non‐participating men) sent the letters refusing to participate in the study. The height, weight, and body mass index of the non‐participating men were similar to the participants (data not shown).

## Discussion

In this study of 1160 young men from general population in Finland and Denmark, we found that semen quality among Danish and Finnish men was remarkably similar. Our results are interesting in relation to a recent meta‐analysis of international data demonstrating an ongoing downward trend in semen quality among Western men, which also includes Finnish men (Levine *et al*., 2017).

Finnish men used to have high sperm concentration at an international scale (Virtanen *et al*., 2013). A study in fertile men in Finland, Scotland, France, and Denmark showed that the median sperm concentration of the men from Finland was the highest, followed by men from Scotland, France and that of the men from Denmark was the lowest (Jørgensen *et al*., 2001). Several young men studies with similar research protocol to the present study were conducted during late 90s to early 2000s (Virtanen *et al*., 2017). The sperm concentration was adjusted to the abstinence period of at least 96 h. Men in Finland and Estonia had higher adjusted sperm concentration than men in Norway and Denmark (54, 57, 41, and 41 million/mL, respectively) (Jørgensen *et al*., 2002). The adjusted sperm concentration of young men reported from Germany was 42–46 million/mL (Paasch *et al*., 2008), Japan, 59 million/mL (Iwamoto *et al*., 2013), Spain, 62 million/mL (Fernandez *et al*., 2012), and Lithuania, 55 million/mL (Punab *et al*., 2002). The recently published study in men from Baltic countries examined during 2003–2004 reported median sperm concentration of 63 million/mL (Erenpreiss *et al*., 2017).

A relatively high level of sperm concentration in Finland was constant during the examination years 1967 to 1994 (Vierula *et al*., 1996). The participants in that study were men from infertile couples. The adjusted sperm concentration in the last year of the study was 85.2 million/ml (Vierula *et al*., 1996). However, this finding does not last any longer, since the 9‐year semen quality trend study in Finland later showed that sperm concentration of the young Finnish men declined from 67 million/mL in examination years 1998–1999 to 48 million/mL in 2006 (Jørgensen *et al*., 2011). In contrast, the 21‐year, prospective, cross‐sectional study in Denmark revealed that there was no persistent trend in the semen quality of the young Danish men during the examination years 1996–2016. Now, our study elucidated that the adverse trend of semen quality in Finland resulted in similar semen quality in Finland and Denmark in terms of sperm production capacity and percentages of spermatozoa with normal morphology. We found slightly better sperm motility in Finland than in Denmark, a finding which should be taken with some caution, as sperm motility evaluations were not coordinated by a quality control system between our two groups.

Etiologies of the declining semen quality among Finnish men are unclear. Since the changes occurred during relatively short period (few years), the causes may be more likely from environmental factors in a broad sense than genetic causes. These may include the man's exposure to the endocrine disrupting chemicals, stress, changing lifestyles, tobacco smoking, marijuana smoking, alcohol consumption, diet, medication intake, or substance use (Barazani *et al*., 2014; Skakkebæk *et al*., 2015; Virtanen *et al*., 2017). The basic clinical characteristics of the Finnish men in our study were relatively similar to the previous study with comparable body mass index, amount of cigarette smoking, and alcohol consumption (Jørgensen *et al*., 2011). Therefore, these factors are probably not the causes of the worsening semen quality in Finland. Subgroup analysis of men who did not take any medications or androgenic steroids, did not have any past or current andrological diseases, or fertility problem resulted in the same conclusion as the analysis of all men. Thus, the reported between‐country differences cannot be explained by these factors. There are a number of genetic defects that are related with lowered semen quality. These include the conditions related with azoospermia or oligozoospermia, such as Klinefelter's syndrome, Y chromosome deletions, Androgen receptor (AR) gene mutation, Cystic fibrosis transmembrane conductance regulator (CFTR) gene mutation, genes related with isolated hypogonadotropic hypogonadism and genes defects found to be associated with reduced sperm motility and abnormal sperm morphology (Hotaling, 2014; Pereira *et al*., 2017; Krausz & Riera‐Escamilla, 2018). However, the incidence of the genetic abnormalities should not change in few years; therefore, genetic abnormalities are unlikely primary etiologies of the declining semen quality.

The role of parental factors and prenatal exposures in determination of semen quality should also be considered. A significant increase in testicular cancer, which most likely has a fetal origin, among Finnish men has been reported (Jørgensen *et al*., 2011) and it seems relevant to speculate that this change and our finding of decreased semen quality may be related to common factors, which interfere with development of the fetal testis (Skakkebæk *et al*., 2001). These factors include fetal exposure to endocrine disrupting chemicals, maternal lifestyle, and health condition during pregnancy. Recently, an association between paternal factors, for example paternal cigarette smoking during pregnancy, and reduced semen quality of the son has also been shown (Axelsson *et al*., 2013). Thus, not only men's current diet, lifestyle, and environmental exposure, but also parental factors and prenatal exposure should be taken into account as possible determinants of semen quality. Cross‐sectional studies can, however, provide only retrospectively collected data on prenatal factors. Such data are prone to recall bias. Therefore, birth cohort studies with prospectively collected prenatal data are necessary to evaluate more carefully the role of these factors on adult male reproductive health.

Sperm concentration less than 40 million/mL relates with impaired fecundity (Bonde *et al*., 1998; Guzick *et al*., 2001; Slama *et al*., 2002). We found that a high proportion of men in our study had sperm concentration below this level both in Finland (38.6%) and Denmark (41.5%). However, it has not been shown which single parameter can best predict fertility, as sperm concentration, sperm morphology, and progressive sperm motility all relate with fertility (Bonde *et al*., 1998; Guzick *et al*., 2001). Therefore, we also reported classes of semen quality when the three semen variables (sperm concentration, the percentage of spermatozoa with normal morphology and progressive motility) all were taken into account, as described previously (Damsgaard *et al*., 2016; Priskorn *et al*., 2018). This is based on the lower reference ranges of WHO and the threshold reported from previous studies that showed the levels of semen variables that are related with fecundity (Bonde *et al*., 1998; Guzick *et al*., 2001; Slama *et al*., 2002). We found that a large proportion of men in Finland (25.4%) and Denmark (34.6%) had poor semen quality, and only 19.3% of men in Finland and 21.5% in Denmark had good semen quality, when all three semen parameters were taken into account (Fig. 1).

It is noteworthy that a large proportion of men in our study did not meet the WHO‐defined lower limits of semen values. These lower reference limits are the 5th centiles of the semen values derived from analysis of several studies in fertile men, whose partners had time to pregnancy of 12 months or less (Cooper *et al*., 2009; World Health Organization, 2010). This is a cause of concern, because poor semen quality relates with increased time to pregnancy and male infertility.

A strength of the present study was the study design and study population. We used the same, standardized protocol for both countries, which allowed us to reliably compare the results. The population in the present study was young men unselected for their general health, education and fertility status. The men and their mothers were born in the country of examination, therefore reducing the variations in ethnicity and geographical environment. Thus, participants may represent general population of the young men in these two Nordic countries, not self‐selected due to fertility problems. The effect of potential confounding factors to semen variables was investigated and adjusted accordingly. Sperm concentration analysis was controlled under external quality control program, except for sperm motility and morphology, see below.

The recruitment of the men in our study was slightly different between Finland and Denmark. In Denmark, the men who undergo medical examination for military service had no known severe or chronic diseases. In Finland, the invitation letters were sent to the young men regardless of health status. However, the questionnaire data revealed that similar proportion of participating men in both countries had particular underlying diseases as shown in Table 1, except more men in Denmark than in Finland had been diagnosed with sexually transmitted disease. Therefore, we believe that the characteristics of the men from the two countries were relatively similar. In addition, the recruitment processes were essentially the same over the years when semen studies were carried out in the two countries.

Our study had some limitations. It is noteworthy that the external quality control was performed for sperm concentration, but not for sperm morphology and sperm motility. In addition, interobserver variation among the clinical examiners between the countries was not studied during the project period. Therefore, the comparison of these semen variables and the clinical examinations between the countries should be interpreted with some caution. In Finland, semen sample collection at home was an alternative. This may increase the time from ejaculation to the start of semen analysis in Finland, which probably decreases sperm motility. Semen volume, sperm concentration, and sperm morphology are not affected. However, in our study, the sperm motility was higher in Finland than in Denmark, even though it might be decreased from the longer time from ejaculation to the start of analysis in Finland. Therefore, the collection of samples at home does not cause major concern for the comparison of semen variables between countries. A common problem for population‐based studies, particularly semen quality studies, is a low participation rate, which may affect the generalization of the results. Comparisons of the characteristics between the participating and non‐participating men might help check the risk of selection bias. The participation rate in Finland was low; therefore, we asked for the basic information of the non‐participants to be compared with the participants. We found that the height, weight, and body mass index reported by the 104 non‐participating men who reported their basic characteristics to the researchers were similar to the participating men. All of them lived in the same areas in Finland as the participants. The age range at invitation was relatively narrow (18–19 years of age), and the men were recruited in a consecutive order according to their date of birth. Therefore, the characteristics of the participating and non‐participating men were rather similar. However, Denmark had higher participation rate than Finland in the present study and the rate was also high for the previous military conscript studies (Andersen *et al*., 2000; Jørgensen *et al*., 2002, 2012; Erenpreiss *et al*., 2017). This may be due to the recruitment methods in Denmark that the invitation to participate in the study was performed by researchers at the site of medical examination for military service. As the data about maternal factors and paternal smoking during pregnancy were retrospectively collected, this information should be interpreted cautiously.

More research to identify causes of deteriorating semen quality is needed. The effect of the endocrine disrupting chemicals needs to be investigated further. In addition, the study of the role of fathers on the sons’ reproductive health through spermatozoa epigenetic mechanisms is necessary (Barratt *et al*., 2018; Donkin & Barrès, 2018).

## Conclusion

In conclusion, we found that semen quality of young Finnish men is converging toward that of Danish men. Our findings are yet another example of deteriorating male reproductive health and parallel to registry studies showing recent increases in testicular cancer among young Finnish men, but rather stable high incidence of testicular cancer in Denmark. Our study did not reveal the causes for this significant finding, which is yet another example calling for much more research in male fertility problems, which contribute significantly to infertility and childlessness.

## Disclosure

The authors have no conflict of interest to declare.

## Authors’ Contributions

N.J., N.E.S., H.E.V., and J.T. designed the study. J.T. is the group leader in Turku center. N.J. is the leader in Copenhagen and also suggested plan for statistical analysis. A.P., S.S. and H.E.V. recruited, examined the men and collected data in Turku. W.R. was responsible for data collection, statistical analysis, sperm morphology assessment (in Turku) and writing the manuscript. All authors were involved in some parts of data analysis and data interpretation. All were involved in revision and approval of the final version of the manuscript.
